# Caregivers’ Self-Rated General Health, Physical and Mental Health Status, Disease Morbidity and Association with Uncontrolled Asthma in Children

**DOI:** 10.3390/pediatric15020023

**Published:** 2023-04-06

**Authors:** Chisa O. Oparanma, Chukwuemeka E. Ogbu, Ebubechukwu Ezeh, Stella C. Ogbu, Otobo I. Ujah, Russell S. Kirby

**Affiliations:** 1 Department of Medicine, Kharkiv National Medical University, 61022 Kharkiv, Ukraine; 2 Chiles Center, College of Public Health, University of South Florida, Tampa, FL 33612, USA; 3 Department of Internal Medicine, Marshall University, Huntington, WV 25701, USA; 4 Department of Biomedical Science, School of Medicine, Tulane University, New Orleans, LA 70112, USA

**Keywords:** caregiver health, child asthma, asthma control, poor mental and physical health, disease morbidity

## Abstract

This study examined the association between caregivers’ self-rated general health, poor physical/mental health days, disease morbidity and asthma control in children from the United States with current asthma. The data analyzed for this study were obtained from 7522 children aged 0–17 years who participated in the 2012–2014, 2015–2017, 2018, and 2019 cycles of the Behavioral Risk Factor Surveillance System Asthma Call-back Survey (ACBS). We employed univariate analysis to describe the study population and weighted binary logistic regression to examine the association of predictors with asthma control. Approximately 50% of the children had uncontrolled asthma. The results show that caregivers who reported fair general health had a 61% higher likelihood of reporting uncontrolled asthma in their children compared to those who reported good/very good/excellent health (adjusted odds ratio [aOR] = 1.61; 95% confidence interval [CI], 1.14–2.26). Poor caregiver general health did not reach statistical significance in predicting uncontrolled asthma (aOR = 1.05, 95% CI, 0.62–1.75). Furthermore, having 1 to 14 poor physical/mental health days ([aOR] = 1.70; 95% CI, 1.28–2.227) and ≥15 poor physical/mental health days (aOR = 1.82, 95% CI, 1.31–2.53) was predictive of uncontrolled asthma in children. Additionally, endorsing one reported disease (aOR = 1.49, 95% CI, 1.15–1.93) and ≥2 diseases (aOR = 1.38, 95% CI, 1.08–1.78) was associated with uncontrolled child asthma. These findings underscore the association between caregivers’ self-reported general health, poor mental/physical health days, disease morbidity and uncontrolled asthma among children from the U.S. with asthma. Pediatricians and child health practitioners should recall the importance of this relationship. To facilitate the identification of caregivers at risk and provide more comprehensive and effective care for children with asthma, healthcare practitioners should utilize every child asthma care encounter to inquire about the overall health of caregivers.

## 1. Introduction

Asthma is one of the most prevalent chronic diseases in children, affecting millions of children worldwide [1]. It is a disease characterized by inflammation and reversible obstruction of the airways, leading to breathing difficulties [2]. Asthma costs the United States economy billions of dollars every year in medical costs, asthma-related mortality, and missed work and school days [2,3]. Therefore, it is no surprise that asthma causes a significant amount of burden to the individual, their family, and society [2]. While conventional therapy has led to a significant global decrease in the morbidity and mortality associated with asthma, some continue to experience symptoms of the condition throughout childhood and adolescence, and even into adulthood [4]. One of the possible reasons is that control of the disease is often dependent on the involvement of the caregiver in the child’s care [5]. Taking care of a child with any chronic illness often takes a demanding amount of emotional and physical responsibility. Performing the role of a caregiver to a child with asthma is no different.

Caregivers play a crucial role in the management of a child’s asthma [6]. They are responsible for ensuring that the child takes their medication, follows a healthy lifestyle, and attends medical appointments [7]. They also identify and remove environmental allergens in the home [7]. However, many caregivers also face numerous challenges in their daily lives, such as poor general, physical, and mental health [8,9,10]. These challenges can affect the caregiver’s ability to provide adequate care for the child, leading to a decline in asthma control. Studies have shown that caregiver general health is positively associated with childhood asthma control [8]. For example, a study of low-income African American families found that caregivers who reported better general health were more likely to provide effective care for their asthmatic children [8]. Poor physical health can lead to fatigue and decreased energy, making them less able to care for their children, including providing support with medication and medical appointments [11]. Moreover, they may be unable to adequately control the home environment, and their children may be exposed to higher levels of allergens and pollution, which can make it more difficult to manage the child’s asthma symptoms [11].

Poor caregivers’ mental health can also have a psychological impact on children. Children whose parents have mental health challenges may experience stress and a feeling of responsibility as a result of their parents’ health problems [12]. This can further exacerbate the child’s asthma symptoms, as stress has been linked to increased airway responsiveness in individuals with asthma [13]. Depression and other psychiatric disorders are common in caregivers of children with asthma [14,15,16,17,18]. Similarly, caregiver’s disease morbidity also influences child asthma control. A study by Kaplan et al. (2020) concluded that the presence of co-morbid conditions in parents can complicate asthma management in children [19]. If caregivers are unable to cope with the child’s demands, they can become subject to abandonment, institutionalization, and overall poor quality of life [20,21,22]. Hence, ascertaining the overall health status of caregivers of children with asthma is very critical to improving the quality of life of children with asthma [23,24,25,26].

The present study, therefore, aimed to examine the association between caregivers’ self-rated general health, physical and mental health, and disease morbidity with the level of asthma control in children. We hypothesize that caregivers who prioritize good general, physical, and mental health and who have a lower disease burden are more likely to have their children’s asthma symptoms under control.

## 2. Materials and Methods

### 2.1. Study Population

Data were obtained from the combined 2012–2014, combined 2015–2017, 2018, and 2019 Behavioral Risk Factor Surveillance System (BRFSS) Asthma Call-Back Survey (ACBS). The BRFSS is a state-based surveillance system that gathers information on chronic diseases, injuries, preventive health practices, health risk behaviors, as well as access and utilization of health care services, including preventable infectious diseases. The Centers for Disease Control and Prevention (CDC) sponsor the BRFSS. The ACBS is a continuous survey conducted two weeks after the BRFSS, which provides yearly data on individuals with asthma, including demographics and medical histories. The survey addresses crucial questions surrounding the health and experiences of people with asthma and provides state- and local-level data. Eligibility for the asthma call-back survey is restricted to respondents who report a prior diagnosis of asthma on the BRFSS. Additionally, if a state includes children in the BRFSS, any randomly selected child who has been previously diagnosed with asthma is eligible for the asthma call-back survey. The Council of American Survey and Research Organizations guidelines are used to calculate response rates for ACBS. The ACBS response rate for states, colonies, and Washington DC participating in the 2012–2019 survey ranged from 36.9 to 93.0% [27].

The dataset used in this study comprised 7522 children who were diagnosed with asthma from 2012 to 2019. Sample survey weights were adjusted in accordance with the recommendations of the ACBS. The ACBS received approval from the Institutional Review Boards of the respective states and the Ethics Review Board of the Asthma and Community Health Branch in the National Center for Environmental Health. Informed consent was obtained from all participants. Since the ACBS is a publicly available dataset, this study is exempt from full institutional review board review. A comprehensive explanation of the data, sampling methods, and other analytical guidelines can be found elsewhere [28].

### 2.2. Outcome Variable: Asthma Control in Children

This study utilized three surrogate measures of asthma impairment, which included daytime symptoms, night-time symptoms, and the use of short-acting β2-agonists (SABA) for symptom control (excluding prevention of exercise-induced bronchospasm), to assess the level of asthma control.

We employed a binary asthma control variable, which comprised two exclusive categories of controlled (day symptoms, ≤2 days a week; Night-time awakenings: ages 0–4 years; ≤1 time a month, ages 5–11 years; ≤1 time a month, ages 12 years or older; ≤2 times a month; Short-acting β2-agonists used for symptom control, ≤2 days a week) and uncon-trolled asthma (day symptoms, >2 days a week; Night-time awakenings: ages 0–4 years; >1 time a month, ages 5–11 years; ≥2 times a month, ages 12 years or older ; >1–3 times a month; Short-acting β2-agonists used for symptom control >2 days a week). This category was based on the 2007 National Asthma Education and Prevention Program Expert Panel Report 3 Guidelines, as the ACBS lacked certain clinical measures required for measuring impairment and future risk assessment of asthma, such as pulmonary function measures, asthma exacerbations, and progressive decline in lung function in children [29]. Previous studies employing the ACBS to assess asthma control have similarly adapted the NAEPP guidelines [30,31].

### 2.3. Predictors: Self-Rated General Health, Poor Physical/Mental Health Days, and Caregiver’s Disease Morbidity

Self-rated general health status of caregivers: The survey item “Would you say that in general your health is…” was used to obtain the caregivers’ self-reported overall health status. The responses were then categorized into three groups: good/very good/excellent, fair, and poor. Earlier studies have established that self-reported health status is a useful tool for predicting an individual’s overall health condition [32].

Caregiver’s poor physical/mental health days: To evaluate the number of days caregivers experienced poor physical or mental health, the survey included an item that asked, “During the past 30 days, for about how many days did poor physical or mental health keep you from doing your usual activities, such as self-care, work, or recreation?” Responses were classified into three categories: none, 1–14 days, and ≥15 days. This measure of poor physical and mental health days has been validated as a reliable measure to track perceived physical and mental health over an extended period [33].

Caregiver’s disease morbidity: The disease morbidity of the caregivers was assessed by adding up the diagnoses of several conditions from the BRFSS questionnaire, including depressive disorder, heart attack, angina/coronary artery disease, stroke, skin cancer, any other cancer diagnosis, diabetes, COPD, arthritis, kidney disease, and morbid obesity. The morbidity status was then classified as having no morbidity, one morbidity, or two or more morbidities. A systematic review has shown that the total number of diseases can predict various health outcomes effectively and perform comparably to more complex measures of disease morbidity [34].

### 2.4. Confounding Variables

Several potential confounding variables were considered, including the child’s age (categorized as 0–4 years, 5–11 years, or ≥12 years), gender (male or female), race (white non-Hispanic, Black non-Hispanic, Hispanic, or multiracial/other non-Hispanic), age at asthma diagnosis (in years), child-reported birth weight (in kilograms), the presence of an asthma action plan (yes or no), the presence of mold in the home (yes or no), caregiver smoking inside the home (yes or no), household income status (categorized as >$75,000, $50,000–$75,000, $25,000–$49,999, or <$25,000), and survey year (ranging from 2012 to 2019).

### 2.5. Statistical Analysis

To improve the representativeness of the U.S. child population with asthma, the ACBS employed a stratified, multistage complex survey design. The analysis of the ACBS data adhered to the recommended analytical guidelines, which incorporated complex analytical procedures. Appropriate weighting was also applied to account for the sampling strata, cluster, and primary sampling unit (PSU) to ensure the accuracy and reliability of the study’s findings.

Bivariate analysis between the covariates and the outcome variable was performed using the Rao-Chi-Square test. A prior literature review was used to select potential confounders, which were then statistically selected through both forward and backward selection. Logistic regression model with weighting was used to regress the covariates against the outcome while controlling for confounders (age of child, birthweight, age of child asthma diagnosis, race, gender, household income, mold in the home, having an asthma management plan, smoking inside the home, and survey year). The results of the weighted logistic regression models were tabulated as odds ratios (ORs) and 95% confidence intervals (CIs), and all percentages were reported as weighted percentages. The absence of multicollinearity between the independent variables was confirmed, as no tolerance estimate fell below 0.1, and no Variance Inflation Factor (VIF) was above 10 [35]. The statistical hypothesis was tested using a two-sided *p* < 0.05 as the level of significance or using non-overlapping 95% confidence intervals. The data were analyzed using SAS 9.4 statistical software (SAS Institute, Cary, NC, USA).

## 3. Results

Among 7522 children respondents with current asthma in our study sample, 3561 (50.4%) reported having uncontrolled asthma (Table 1).

The proportion of children who reported uncontrolled asthma was significantly greater than the proportion who reported controlled asthma among the following groups: females (52.5% vs. 47.5%), children aged 0–4 years (52.4% vs. 47.6%), children aged 5–11 years (56.1% vs. 43.9%), respondents of Black non-Hispanic race/ethnicity (59.2% vs. 40.8%), respondents of Hispanic ethnicity (58.1% vs. 41.9%), children in the household with income $25,000–$49,999 (51.5% vs. 48.5%), children in the household with income <$25,000 (63.5% vs. 36.5%), caregivers who endorse fair health (62.1% vs. 37.9%), caregivers who endorse poor health (61.7% vs. 38.3%), caregivers who reported 1–14 poor physical/mental health days (58.9% vs. 41.1%), %), caregivers who reported ≥15 poor physical/mental health days (65.2% vs. 34.8%), caregivers who endorsed one disease (52.5% vs. 47.5%), and two diseases (50.1% vs. 49.9%).

In the weighted logistic regression model, caregivers who reported fair health status had a 61% higher likelihood of reporting uncontrolled asthma in their children compared to those who reported good, very good, or excellent health (adjusted odds ratio [aOR] = 1.61; 95% CI, 1.14–2.26). However, poor caregiver general health did not reach statistical significance in predicting uncontrolled asthma (aOR = 1.05, 95% CI, 0.62–1.75). Caregivers who reported 1–14 poor physical/mental health days were 70% more likely to have children with uncontrolled asthma compared to caregivers with no reported poor physical/mental health days (aOR = 1.70; 95% CI, 1.28–2.27) (Table 2).

Having ≥15 poor physical/mental health days was associated with 82% increased odds of uncontrolled asthma in children (aOR = 1.82, 95% CI, 1.31–2.53). Caregivers with one reported disease were 1.5 times more likely to report uncontrolled asthma in their children (aOR = 1.49, 95% CI, 1.15–1.93) while the odds of uncontrolled child asthma were higher among caregivers with two or more disease conditions (aOR = 1.38, 95% CI, 1.08–1.78).

## 4. Discussion

The aim of this study was to examine the association between caregivers’ self-rated general health, mental/physical health status, disease morbidity, and asthma control in children. The descriptive analysis indicated that the frequency of uncontrolled asthma differed based on age, race, household income, caregiver’s general health, poor physical/mental health days, and caregiver’s disease burden. These results are in agreement with previous studies that have reported similar findings [9,10,11,12,17,26].

The adjusted multivariate analysis indicated that the association between caregivers’ self-rated health and the likelihood of uncontrolled asthma weakened after adjusting for confounders in caregivers who reported poor health status. However, caregivers reporting fair health status had a positive association with uncontrolled asthma. These mixed findings are contrary to previous studies that demonstrated a positive correlation between caregiver health and asthma control [14,15,16,17]. The inconsistency in the results could be attributed to the small sample size of caregivers who reported poor health status.

A significant association was observed between the number of poor mental/physical health days and uncontrolled asthma. This result was anticipated since prolonged physical illness periods for caregivers can reduce the time available to manage the child’s asthma symptoms. Moreover, studies indicate that children with physically ill parents may experience stress, anxiety, guilt, or responsibility for their parents’ health, exacerbating the child’s asthma symptoms, as stress has been linked to heightened airway responsiveness in individuals with asthma [12,13]. Easter et al. (2015) conducted a meta-analysis that showed that caregivers of children with asthma had a higher prevalence of depression and anxiety than caregivers of healthy children [14]. Ortega et al. (2004) also found that Puerto Rican children with parents experiencing mental health issues were more likely to report histories of asthma attacks [15]. In addition, Brown et al. (2006) reported that caregiver depression was associated with unscheduled child asthma clinic visits and asthma-related hospitalizations [16]. Therefore, mental health, like physical health, can impact the caregiver’s ability to focus on the child’s care [17], leading to stress and financial challenges, which can increase the likelihood of poor health behaviors such as smoking, ultimately exacerbating the child’s asthma symptoms [18].

The analysis revealed a notable increase in the likelihood of uncontrolled asthma in children whose caregivers suffered from one, two, or more diseases, compared to those whose caregivers did not experience any morbidity. These results are in accordance with Kaplan et al.’s (2020) research that established a significant correlation between parental comorbidities and asthma management in children. Additionally, the caregiver’s disease burden can affect the household’s general quality of life, which ultimately has a direct impact on asthma control, as highlighted by previous studies [20,21,22].

Various strategies can be used to reduce the impact of caregiver health on the control of asthma in children. Firstly, it is essential to address the underlying causes of poor physical and mental health in caregivers. This may include providing access to healthcare, addressing financial concerns, and addressing social determinants of health that can contribute to poor health and well-being [7,8]. By addressing the root causes of poor health in caregivers, it is possible to improve the health and well-being of caregivers and the children in their care [11,12].

An indispensable approach involves imparting knowledge and providing resources to children and their caregivers by pediatricians, child nurses, and child public health experts to enhance the management of asthma. Such interventions may include educating children on identifying and addressing early signs of asthma, emphasizing the significance of adhering to medication regimens, and offering guidance on mitigating exposure to asthma triggers [13]. Moreover, utilizing every child healthcare encounter as an avenue to inquire about the overall health of caregivers is an additional strategy that could be employed. This inquiry could aid in the identification of caregivers who may be experiencing poor general, physical, or mental health. Subsequently, the information gathered could be utilized to develop interventions geared towards improving asthma control and enhancing the quality of life of children with asthma. This approach could enhance the ability of pediatricians and other child health practitioners to provide comprehensive and effective care to children with asthma [36].

This study has notable strengths. The authors utilized the Asthma Call-Back Survey (ACBS) dataset, which includes a nationally representative sample of non-institutionalized children in the United States. This allowed for the estimation of asthma prevalence across diverse subgroups of the population with greater accuracy and precision. Additionally, the large sample size of the ACBS dataset provided ample opportunity to explore the relationship between asthma control and a range of self-reported health outcomes, as well as the mental and physical health of caregivers, while accounting for potential confounding factors that may affect the association between exposure and outcome.

Several limitations should be acknowledged in this study. Firstly, as the ACBS is a cross-sectional survey, it is not possible to establish a causal relationship between asthma control and predictors. Furthermore, institutionalized, and hospitalized children were not interviewed in the ACBS, which could potentially underestimate the prevalence of severe and active asthma and limit the generalizability of the findings. The study is also limited to households that could be reached by phone. Another limitation is the self-reported nature of both the child’s asthma symptoms and the caregiver’s health status, which introduces the possibility of recall bias and misclassification. The absence of clinical measures, such as pulmonary function tests, and information on future risk assessment in the ACBS may result in differential misclassification bias of the outcome measure (asthma control).

## 5. Conclusions

The results of this study underscore the association between caregivers’ self-reported general health, poor mental/physical health, disease morbidity, and uncontrolled asthma in children from the U.S. with current asthma. It is crucial for pediatricians and other child health practitioners to bear in mind this relationship. Further studies on asthma in children should continue to longitudinally explore the association of asthma control patterns and caregivers’ health and psychosocial function, as this could improve a child’s asthma-related quality of life and contribute towards achieving the 2030 Healthy People objective of adequate respiratory health. To facilitate the identification of caregivers at risk and to provide more comprehensive and effective care for children with asthma, healthcare practitioners should utilize every child asthma care encounter to inquire about the overall health of caregivers.

## Figures and Tables

**Table 1:** Descriptive statistics of caregiver’s self-rated health-related quality of life and uncontrolled asthma among. children from the U.S: Behavioral Risk Factor Surveillance System Asthma Call-back Survey, 2012–2019.

Characteristics			Asthma Control			
	Survey Respondents		Uncontrolled		Controlled	
	No, ^a^	% ^b^ (95% CI ^b^)	No, ^a^	% ^b^ (95% CI ^b^)	No, ^a^	% ^b^ (95% CI ^b^)
**Total**	7522		3561	50.4 (48.1–52.7)	3961	49.6 (47.3–51.9)
**Gender**						
Female	3147	43.1 (40.7–45.4)	1514	52.5 (48.9–56.1)	1633	47.5 (43.9–51.1)
Male	4375	56.9 (54.6–59.3)	2047	48.9 (45.8–52.0)	2328	51.1 (48.0–54.2)
**Age** ^c^						
0–4 years	1235	29.7 (27.5–31.8)	654	52.4 (47.4–57.3)	581	47.6 (42.7–52.6)
5–11 years	2705	37.0 (34.9–39.2)	1378	56.1 (52.4–59.8)	1327	43.9 (40.2–47.6)
≥12 years	3582	33.3 (31.4–35.1)	1529	42.4 (39.3–45.5)	2053	47.6 (54.5–60.7)
**Race/ethnicity** ^c^						
White non-Hispanic	5539	65.3 (63.0–67.6)	2499	47.3 (44.6–49.9)	3040	52.7 (50.1–55.4)
Black non-Hispanic	865	20.2 (18.1–22.3)	505	59.2 (52.9–65.6)	360	40.8 (34.4–47.1)
Multiple/Other race non-Hispanic	735	10.6 (9.1–12.1)	359	48.1 (40.1–56.2)	376	51.9 (43.8–59.9)
Hispanic	230	3.9 (3.0–4.8)	122	58.1 (45.6–70.6)	108	41.9 (29.4–54.4)
**Household income** ^c^						
>$75,000	2859	36.7 (34.5–38.8)	1151	41.9 (38.3–45.4)	1708	58.1 (54.6–61.7)
$50,000–$75,000	1089	13.8 (12.3–15.3)	467	39.6 (33.8–45.3)	622	60.4 (54.7–66.2)
$25,000–$49,999	1336	17.9 (16.2–19.5)	698	51.5 (46.4–56.7)	638	48.5 (43.3–53.6)
<$25,000	1807	31.7 (29.3–34.1)	1044	63.5 (59.0–68.0)	763	36.5 (32.0–41.0)
**Caregiver’s self-rated general health** ^c^						
Good/very good or excellent	6196	80.6 (78.6–82.5)	2798	47.7 (45.2–50.1)	3398	52.3 (49.9–54.8)
Fair	1000	15.1 (13.3–16.9)	555	62.1 (56.2–68.1)	445	37.9 (31.9–43.8)
Poor	306	4.3 (3.3–5.3)	198	61.7 (49.3–74.1)	108	38.3 (25.9–50.7)
**Poor physical/mental health days** ^c^						
None	2564	54.0 (51.3–56.7)	1167	47.1 (43.2–51.0)	1397	52.9 (49.0–56.8)
1–14 days	1456	32.7 (30.1–35.3)	784	58.9 (54.1–63.8)	672	41.1 (36.2–45.9)
≥15 days	670	13.3 (11.6–15.0)	420	65.2 (58.6–71.8)	250	34.8 (28.2–41.4)
**Have an asthma action plan**						
No	3435	47.0 (44.6–49.5)	1591	49.8 (46.3–53.3)	1844	50.2 (46.7–53.7)
Yes	3958	53.0 (50.5–55.4)	1911	51.5 (48.3–54.7)	2047	48.5 (45.3–51.7)
**Caregiver disease morbidity** ^c^						
None	2227	30.6 (28.4–32.7)	943	43.6 (39.7–47.6)	1284	56.4 (52.4–60.3)
One disease	2343	32.0 (29.7–34.3)	1074	45.8 (48.1–56.8)	1269	54.2 (43.2–51.8)
≥Two diseases	2952	37.4 (32.1 –40.1)	1544	52.3 (44.5–55.7)	1408	47.7 (44.3–55.5)
**Mold in home**						
No	6917	91.7 (90.4–93.0)	3216	49.9 (47.5–52.3)	3701	50.1 (47.7–52.5)
Yes	581	8.3 (7.0–9.6)	332	57.3 (48.8–65.8)	249	42.7 (34.2–51.2)
**Smoking inside home**						
No	7089	94.7 (93.8–95.7)	3310	49.8 (47.4–52.2)	3701	50.2 (47.8–52.6)
Yes	421	5.3 (4.3–6.2)	245	62.6 (54.2–71.0)	249	37.4 (29.0–45.8)

CI, confidence interval. ^a^ Unweighted pooled sample size, 2012–2019. Due to item non-response, individual characteristic categories may not sum to total. ^b^ Weighted prevalence and 95% confidence interval. ^c^ *p* Values < 0.05 for the chi-square test of association between asthma control and all selected variables.

**Table 2:** Multivariable logistic regression of caregiver’s self-rated health-related quality of life and uncontrolled asthma among children from the U.S.: Behavioral Risk Factor Surveillance System Asthma Call-back Survey, 2012–2019.

Model	Unadjusted OR (95% CI) ^b^	Adjusted OR (95% CI) ^b^
**Caregiver’s self-rated general health** ^a^		
Good, very good or excellent	Ref	Ref
Fair	1.80 (1.35–2.40)	1.61 (1.14–2.26)
Poor	1.77 (1.01–3.11)	1.05 (0.62–1.75)
**Poor physical/mental health days** ^a^		
None	Ref	Ref
1–14 days	1.61 (1.21–2.14)	1.70 (1.28–2.27)
≥15 days	2.11 (1.40–3.16)	1.82 (1.31–2.53)
**Caregiver disease morbidity** ^a^		
None	Ref	Ref
One disease	1.43 (1.14–1.83)	1.49 (1.15–1.93)
≥Two diseases	1.54 (1.22–1.95)	1.38 (1.08–1.78)

CI, confidence interval; OR, Odds Ratio. ^a^ Each predictor was separately adjusted for age of child, birthweight, age of child asthma diagnosis, race, gender, income status, mold in the home, having an asthma management plan, smoking inside the home, and survey year. ^b^ Weighted OR and 95% confidence interval.

## Data Availability

The data used to generate the findings of this study are publicly available in the CDC Asthma Call-Back Survey Website available at: https://www.cdc.gov/brfss/acbs/index.htm (accessed on 9 February 2023). The Asthma Call-back Survey (ACBS) is a product of the CDC’s National Asthma Control Program (NACP).
